# Low-frequency rTMS in patients with subacute ischemic stroke: clinical evaluation of short and long-term outcomes and neurophysiological assessment of cortical excitability

**Published:** 2015

**Authors:** AV Blesneag, DF Slăvoacă, L Popa, AD Stan, N Jemna, F Isai Moldovan, DF Mureșanu

**Affiliations:** * Department of Clinical Neurosciences, ‘‘Iuliu Hațieganu’’ University of Medicine and Pharmacy, Cluj-Napoca, Romania; ** ‘‘RoNeuro’’ Institute for Neurological Research and Diagnostic, Cluj-Napoca, Romania; *** County Emergency Hospital, Cluj-Napoca, Romania

**Keywords:** subacute ischemic stroke, low-frequency rTMS, cortical excitability

## Abstract

**Rationale:** Repetitive transcranial magnetic stimulation (rTMS) is used alone or in combination with physiotherapy for rehabilitation of stroke patients. TMS mapping can also quantify the excitability of the motor area in both the ipsilesional (IL) and contralateral (CL) hemisphere.

**Objective:** This study is the first to measure the dynamics of cortical excitability by TMS mapping before and after treatment with low-frequency (LF) rTMS in the contralesional hemisphere at three different timepoints. Furthermore, the patients were clinically evaluated during the same visit as the mapping to establish both short and long-term outcomes after rTMS treatment.

**Methods and Results:** A total of 16 participants with acute ischemic stroke were assessed 10 days post-stroke by TMS mapping. The patients were randomized into two equal groups: a real rTMS group and a sham group. The rTMS group received LF-rTMS to the contralesional hemisphere for 10 days, starting on the first day after the first mapping. Each subject was also evaluated by mapping on days 45 and 90 after stroke onset. The primary clinical outcome measured was the Fugl-Meyer Assessment for Upper Extremity (FMA-UE) on days 10, 45 and 90 post-stroke. At 10 days after stroke onset, both groups presented low excitability in the lesion side and high excitability in the non-affected side. In the real rTMS group, at 45 days after stroke, a downward trend in the excitability of the contralesional hemisphere and an upward trend in the excitability of the lesioned side were observed. At 90 days after stroke, a tendency toward balanced excitability between both hemispheres was observed. In the sham group, at both 45 and 90 days, we observed increased excitability in the non-affected side compared to the side with the lesioned motor area. At 45 days, the real rTMS group demonstrated a better recovery of the upper limb motor function than the sham group, but at 90 days, there was no significant difference between the two groups.

**Discussion:**These results demonstrated that LF-rTMS treatment enhances rebalance of the excitability patterns in both hemispheres and led us to question the “one size fits all” approach widely used in rTMS interventions.

**Abbreviations:** Amax = maximum amplitude, Amean = AM = averaged amplitude, APB = abductor pollicis brevis, CL = contralesional, DTI = diffusion tensor imaging, EEG = electroencephalography, EMG = electromyography, FMA-UE = Fugl-Meyer Assessment for Upper Extremity, HS = hot spot, IHC = interhemispheric functional connectivity, IL = ipsilesional, LF-rTMS = low-frequency repetitive transcranial magnetic stimulation, MCA = middle cerebral artery, MEP(s) = motor evoked potential(s), NIBS = non-invasive brain stimulation, rMT = resting motor threshold, RP = responsive points, rTMS = repetitive transcranial magnetic stimulation, TMS = transcranial magnetic stimulation

## Introduction

Currently, arterial recanalization is the only approved specific treatment for acute ischemic stroke [**1**]. Despite the fact that a large number of clinical studies have focused on pharmacological and non-pharmacological interventions aimed at providing neuroprotection and enhancing neurorecovery, the results of these studies are controversial [**2**]. Of the non-pharmacological interventions, non-invasive brain stimulation (NIBS) has been highlighted as a promising approach, but the results from clinical studies of NIBS are far from conclusive. To achieve better results with NIBS, it is essential to understand the dynamics of the connectomics that occur after stroke, the way NIBS interferes with neuroplasticity, and the way these mechanisms influence recovery after stroke.

Computational models, neuroimaging and EEG studies have revealed changes in the overall connectivity of the brain after stroke, as a consequence of both intra- and interhemispheric structural and functional reorganization. Structural reorganization is based on the structural plasticity of synapses, dendrites and axons and has direct consequences related to the rewiring of brain networks the induction of specific patterns of synchronized/ desynchronized neural activity across widespread areas, including in the contralesional (CL) hemisphere [**3**-**5**]. In a study of animal models, van Meer et al. showed that there is a decrease in intraregional coherence and interhemispheric functional connectivity after medium and large strokes. Graph-based network analyses showed an increased clustering coefficient, shortest path length, and small-worldness in the bilateral sensorimotor cortex, probably as a consequence of the hyper-connectivity in the lesion’s surrounding areas and in the contralateral hemisphere [**6**]. However, other studies that investigated overall brain connectivity, from the point of view of graph theory, showed a general reduction of small-worldness, as a consequence of disruptions to long-distance connections [**7**,**8**]. These apparently conflicting results suggest that there is an imbalance between local specialization and global integration. This idea is in concordance with the concept of “diaschisis”, which was introduced by Constantin von Monakow in 1914 and which in its newer and broader sense indicates that neurological deficits are not just a consequence of the lesion itself but also are related to the secondary effects of the lesion in related areas, effects that have repercussions throughout the entire brain network [**9**-**12**].

The imbalance in interhemispheric functional connectivity includes an increase in the excitability of the contralesional hemisphere, which has been described in both basic research and clinical studies [**13**-**15**]. This hyper-excitability was explained by the recruitment of circuits normally involved in other functions through the unmasking of the uncrossed fibers to the paretic arm/ leg and reduced transcallosal inhibitory pathways. The rivalry theory is based on the idea that contralesional over-activation itself has an inhibitory effect on the lesioned hemisphere and that persons with a prolonged presentation of this pattern may experience worse long-term outcomes [**16**-**18**]. It appears that the hyper-activation of the unaffected hemisphere is responsible for sustained mirror movements during the movement of the paretic limb; these mirror movements are considered to be detrimental to the neurorehabilitation process [**19**]. On the other hand, other studies have shown that perilesional reorganization alone might not be sufficient for the functional recovery, especially in cases of medium and severe strokes, and that there is a need for contralesional hemisphere overactivation to compensate for the neuronal loss. This is referred to as the vicariation model [**20**,**21**]. Even if the subject remains under debate, low-frequency repetitive transcranial magnetic stimulation (LF-rTMS) has been intensively applied in clinical trials to inhibit the non-lesioned hemisphere in patients with motor impairment or aphasia. A meta-analysis from 2014 and a systematic review from 2015 suggested that LF-rTMS on the ipsilesional (IL) hemisphere has a positive effect, but more studies in this direction are needed [**22**,**23**].

Single pulse transcranial magnetic stimulation (TMS) was used to investigate differences in cortical excitability and the broadening of motor area reorganization after stroke. People with motor deficits post-stroke usually presented reduced amplitude motor-evoked potentials (MEPs) on the affected side compared with the unaffected motor area. Higher MEPs on the affected side were correlated with better clinical outcome [**24**-**26**]. During one year of serial evaluations, starting on the first day of stroke, Delvaux et al. showed the MEPs in the non-affected side were characterized by higher amplitudes in the early evaluation than in later evaluations of the same side, but that they remained higher than on the lesioned side even one year after stroke [**26**]. Several LF-rTMS studies that have referred to MEP measurements before and immediately after the treatment have shown a decrease in MEP amplitudes in the contralateral hemisphere immediately after the rTMS sessions [**27**,**28**].

The present study examined the dynamics of TMS mapping parameters between the two hemispheres before and after LF-rTMS or sham intervention on the CL motor area, from the point of view of interhemispheric imbalance. We also analyzed motor function outcomes in relation to rTMS intervention. 

## Materials and methods

**Participants**

Patients were recruited from the Neurological Department of the Cluj-Napoca County Emergency Hospital. The inclusion criteria were: age > 18 years; 10 days from stroke onset; ischemic stroke in the region of the medium carotid artery (MCA) with motor deficit at the level of the upper limb; first-ever stroke; and ability to understand and submit to the treatment and evaluation. Exclusion criteria were pregnancy, cardiac pacemaker, medical history of seizure, aneurysm clip, traumatic brain injury or other neurological disorders, or other medical serious complications, such as pneumonia and severe heart failure. In total, 16 stroke patients were randomized into 2 equal groups: the real rTMS group consisted of 8 patients, and the sham group also consisted of 8 patients. Participants in both groups were provided a written informed consent, which was approved by the Ethics Committee of “Iuliu Hațieganu” University of Medicine and Pharmacy Cluj-Napoca.

**Study Design**

This is a prospective, randomized, placebo-controlled, single-blind clinical study, that consisted of the following visits: 

• Visit 1 (baseline) at 10 days from stroke onset: demographic data, inclusion/ exclusion criteria, upper limb motor function evaluation by Fugl-Meyer Assessment for Upper Extremity (FMA-UE), first TMS mapping. rTMS/ sham stimulation was applied once per day, 5 days/ week, for 10 working days;

• Visit 2 at 45 days from stroke onset: FMA-UE, the second TMS mapping;

• Visit 3 at 90 days from stroke onset: FMA-UE, the third TMS mapping.

**rTMS intervention**

A MagPro X100 device (MagVenture, Denmark) with a figure-8 coil (C-B60) was used for repetitive stimulation. We applied 10 consecutive sessions (excluding weekends) of 20 min using 1 Hz rTMS/ day on the non-lesioned M1. The coil was placed tangentially to the scalp, with the handle pointing 45° postero-laterally, on the hot spot (HS) of the unaffected abductor pollicis brevis (APB) motor area. Each rTMS consisted of 1200 pulses with a stimulus intensity of 120% of the resting motor threshold (rMT). Sham stimulation was given by positioning the coil at the same location but with an intensity of 10% of the resting motor threshold (rMT), which provided a skin sensation similar to real stimulation [**29**].

**Outcome measures**

**Fugl-Meyer Assessment for the Upper Extremity**

FMA-UE was chosen as the primary clinical outcome measure and was performed by blinded evaluators. This test has been established as reliable and valid and has also been used extensively to evaluate the upper extremity motor function in other clinical trials [**30**,**31**]. It consists of 33 items, and each item is rated on a three-point ordinal scale (0 = cannot, 1 = can perform partially, and 2 = can perform fully), with the maximum motor performance score for the upper limb being 66 points [**32**].

**Neurophysiological Assessment (TMS mapping)**

Each subject sat in a comfortable chair and wore a helmet with equal marks 1 cm apart. We recorded MEPs from the contralateral APB muscle. We used bipolar adhesive monitoring electrodes (H59P, Kendall soft-ETM, Chicopee, MA) and placed the active electrode on the belly of the muscle and the reference electrode placed proximally, at a distance of 1.5 cm. The electromyographic (EMG) signals were filtered by CareFusion Nicolet EDX, with a 2 Hz-10 KHz filter, setting and amplified in 4 channels of a Nicolet AT2+6 with Viking EDX software. TMS was delivered by a MagPro X100 (MagVenture, Denmark) stimulator by using a C-B60 coil. The mapping was performed on both hemispheres. The coil was placed tangential to the scalp with the handle pointing 45° antero-medially. We identified the HS of the motor area by successive stimulations at 100% intensity at every point of a 4 cm x 4 cm grid, with the posterior-medial corner of the grid being situated at 2 cm lateral to the vertex and 1 cm anterior. The HS was marked with a marker on the helmet. Second, we identified the motor threshold intensity of the HS. The stimulation intensity was progressively increased until the rMT was reached. This was performed by eliciting reproducible MEPs – at least 50 µV in amplitude – in 5 of 10 consecutive stimuli [**33**,**34**]. Afterwards, we determined the MEP amplitude in the HS after stimulation with 120% intensity of the rMT. We also determined the MEP amplitudes of adjacent positions. If another point had a higher amplitude than HS, we began the procedure again and considered that point to be the HS [**35**]. The primary motor area of the target muscle was mapped by repeated stimulations, centimeter by centimeter, over 32 points (a total of 33 points with HS): 4 points each in the antero-posterior axis and medio-lateral axis and 4 points square in the antero-medial, antero-lateral, postero-medial, and postero-lateral axes. Four consecutive MEPs, with a repetition rate of 0.1-0.2c/ s and with 120% rMT, were gathered from each grid position, and each position was recorded to obtain the highest amplitude. In total, 132 stimuli were applied to each hemisphere, and the entire mapping session duration was of approximately 60 min.

**Data analyses**

We measured the following parameters of cortical excitability for both hemispheres: the maximum amplitude (Amax) that we obtained with 100% intensity (the one that determined the HS), the rMT, the number of responsive points (where the amplitude was higher than 50 µV) (RP), and the overall averaged amplitude obtained with 120% intensity (AM).

The main statistical method used was a 2 by 2 repeated measures multivariate analysis of variance (MANOVA), which was used to assess changes in cortical excitability in the hemisphere and group at multiple time points [within group independent variable: type of hemisphere (affected-unaffected) and between subjects independent variable: Groups (rTMS-sham); dependent variable: scores of cortical excitability parameter of interest was measured at 3 time points: V1-V2-V3]. A post hoc analysis (t test) was used to identify statistically significant main effects or interaction effects.

The repeated measures ANOVA was used to test: 1) the changes in the mean FMA-UE scores over three time points, 2) differences in mean scores between rTMS and sham patients and 3) if the pattern in mean differences of FMA-UE scores depends upon the group type (rTMs or sham).

The level of statistical significance for all two-sided tests was set at p < 0.05. 

Statistical analyses were performed with R, advanced software environment for statistical computing and graphics, version 3.1.2 (R Foundation for Statistical Computing, Vienna, Austria).

## Results

The real rTMS group had the following characteristics: 6 male/ 2 female; mean age 69 ± 5.8 years, range 62-76 years; all right-handed; and 6 right- and 2 left-hemispheric lesions. The sham rTMS group had the following characteristics: 4 male/ 4 female and mean age 69.13 ± 7.2 years, range 53-79 years. We did not observe a difference in patient age (t-test on independent groups with equal variance, t=0.032, df=14, p=0.098). In the real rTMS group, 6 participants had right- and 2 had left-hemispheric lesions. In the sham rTMS group, 4 persons had right- and 4 had left-hemispheric lesions. All volunteers were right-handed.

**Change in Fugl-Meyer Assessment Upper Extremity scores**

There was a statistically significant effect of time on FMA-UE scores [ANOVA repeated measures, Pillai’s Trace; V=0.943; F (2.13)=107.99; p<0.001]. A significant interaction was found between time (V1-V2-V3) and group [F (2.13)=4.30; p=0.037]. The post hoc analysis used to locate the source of interaction revealed significant changes in FMA-UE scores from V1 to V2 (paired t-test, t=5.85; df=7; p=0.001) and from V1 to V3 (paired t-test, t=11.18; df=7; p<0.001) in rTMS patients. An improvement was observed in FMA-UE mean scores in the rTMS group between V1 and V2 timepoints (mean ± standard deviation: 29.63 ± 12.65 versus 42.88 ± 16.81) and between V1 and V3 (29.63 ± 12.65 versus 45.00 ± 13.40). From the V2 to V3 timepoints, there was an increase in the mean score without statistical significance (paired t-test; t=1.54; df=7; p=0.168). In the sham rTMS group, a significant change was observed in FMA-UE mean scores between any two time points: from V1 to V2 (t=4.03; df=7; p=0.005), from V1 to V3 (t=9.94; df=7; p<0.001), and from V2 to V3 (t=5.24, df=7; p=0.001).

At baseline (V1), there was no significant difference in FMA-UE mean scores between the two groups of participants (t-test for independent groups with equal variances t=0.34; df=14; p=0.74). Also, there was no significant difference in FMA-UE mean scores between the two groups at the V2 timepoint (t=0.60; df=14; p=0.56) or 90 days (t=0.32; df=14; p=0.76).

**Fig. 1:**
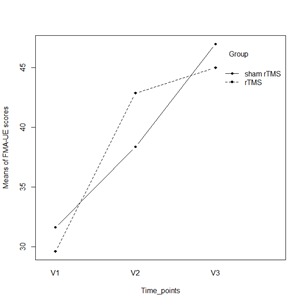
Mean plot of FMA-UE scores over time and between groups

**Neurophysiological assessment by mapping**

**Dynamic changes in rMT parameter**

Repeated measures MANOVA showed that there was a significant, multivariate and hemisphere effect [Pillai’s Trace multivariate test: V=0.64; F(3,12)=7.04; p=0.006]. Univariate analysis showed that was also a significant hemispheric effect on rMT values at each time point (p<0.05), regardless of the subject’s group. Within-group univariate analysis revealed that rMT values at V1 [Greenhouse-Geisser correction F (1,14)=16.548; p=0.001], rMT values at V2 [Greenhouse-Geisser correction F(1,14)=6.189; p=0.026] and rMT values at V3 [Greenhouse-Geisser correction F(1,14)=4.783; p=0.046] were significantly different between the two hemispheres. According to MANOVA, the effect of group was statistically insignificant [V=0.24; F(3,12)=1.24; p=0.337], while there was a trend toward significance in the hemisphere × group interaction [V=0.40; F(3,12)=2.63; p=0.098]. The source of this interaction was due to differences in variability of rMT scores at the V2 timepoint between the two groups [Greenhouse-Geisser correction F (1,14)=3.41; p=0.086]. Only in the real rTMS group were the values of the rMT parameter measured in the IL hemisphere lower than the rMT of the CL (**Table 1**), with a tendency toward statistical significance (paired t-test; t=2,22; df=7; p= 0.062).

**Table 1:** Cortical excitability measures for rMT parameters in the two groups and hemispheres over three visits

Measure	Time points	Group	Hemispheres	Mean scores	SE	95% CI
rTM	V1	rTMS	IL	63,25	5,50	51,45-75,05
			CL	52,50	5,61	40,46-64,54
		sham	IL	74,38	5,50	62,57-86,18
			CL	62,38	5,61	50,33-74,42
rTM	V2	rTMS	IL	65,88	3,86	57,59-74,16
			CL	79,38	4,84	68,99-89,75
		sham	IL	65,50	3,86	57,22-73,78
			CL	67,50	4,84	57,12-77,88
rMT	V3	rTMS	IL	68,00	5,95	55,25-80,75
			CL	69,88	5,73	57,59-82,16
		sham	IL	66,63	5,95	53,88-79,38
			CL	72,13	5,73	59,84-84,41
*SE=standard error; CI=confidence interval (lower limit-upper limit)*						

**Dynamic changes in RP parameter**

Repeated measures MANOVA showed that there was a significant, multivariate and hemisphere effect [Pillai’s Trace multivariate test: V=0.60; F (3,12)=6.06; p=0.009]. Univariate analysis showed that there was also a significant hemisphere effect on RP values at the V1 timepoint (p<0.05), regardless of the subject’s group. Within-group univariate analysis revealed that the RP values at V1 [F(1,14)=12.67; p=0.003] were significantly different between the two hemispheres and that the RP scores at V2 [F(1,14)=0.001; p=0.972] and the RP values at V3 [F(1,14)=1.76; p=0.205] were not significantly different between the two hemispheres. According to MANOVA, the effect of group was statistically insignificant [V=0.15; F(3,12)=0.69; p=0.573], while the hemisphere × group interaction was significant [V=0.51; F(3,12)=4.09; p=0.032]. The source of this interaction was the differences in variability in the RP scores between the two groups at the V2 timepoint [F(1,14)=6.70; p=0.021] and the V3 time point [F(1,14)=12.04; p=0.004]. Only in the real rTMS group did we observe a significant difference in mean values of RP parameters measured on IL and CL (paired t-test; t=2.61; df=7; p=0.035), while in the sham group, there was no significant difference (paired t-test; t=1.50; df=7; p= 0.176). The mean RP IL value at the V2 timepoint was greater than the mean RP CL value of the rTMS group (**Table 2**).

**Table 2:** Cortical excitability measures of RP parameters in the two groups and hemispheres over three visits

Measure	Time points	Group	Hemispheres	Mean scores	SE	95% CI
RP	V1	rTMS	IL	16,75	1,26	14,04-19,46
			CL	19,13	1,60	15,70-22,55
		sham	IL	14,88	1,26	12,17-17,58
			CL	19,0	1,60	15,58-22,42
RP	V2	rTMS	IL	22,88	1,66	19,32-26,43
			CL	18,38	1,694	14,74-22,01
		sham	IL	18,13	1,66	14,57-21,68
			CL	22,75	1,69	19,12-26,38
RP	V3	rTMS	IL	23,88	1,77	20,09-27,67
			CL	20,75	1,93	16,61-24,90
		sham	IL	15,88	1,76	12,09-19,67
			CL	22,88	1,93	18,73-27,02
*SE=standard error; CI=confidence interval (lower limit-upper limit)*						

In the sham rTMS group, at 90 days (V3), there was a significant difference in mean values (paired t-test; t=3.03; df=7; p= 0.019) and we observed that the mean RPV3 in the CL hemisphere was greater than the RPV3 measured in the IL hemisphere (**Table 2**). 

**Dynamic changes in AM parameter**

**Fig. 2:**
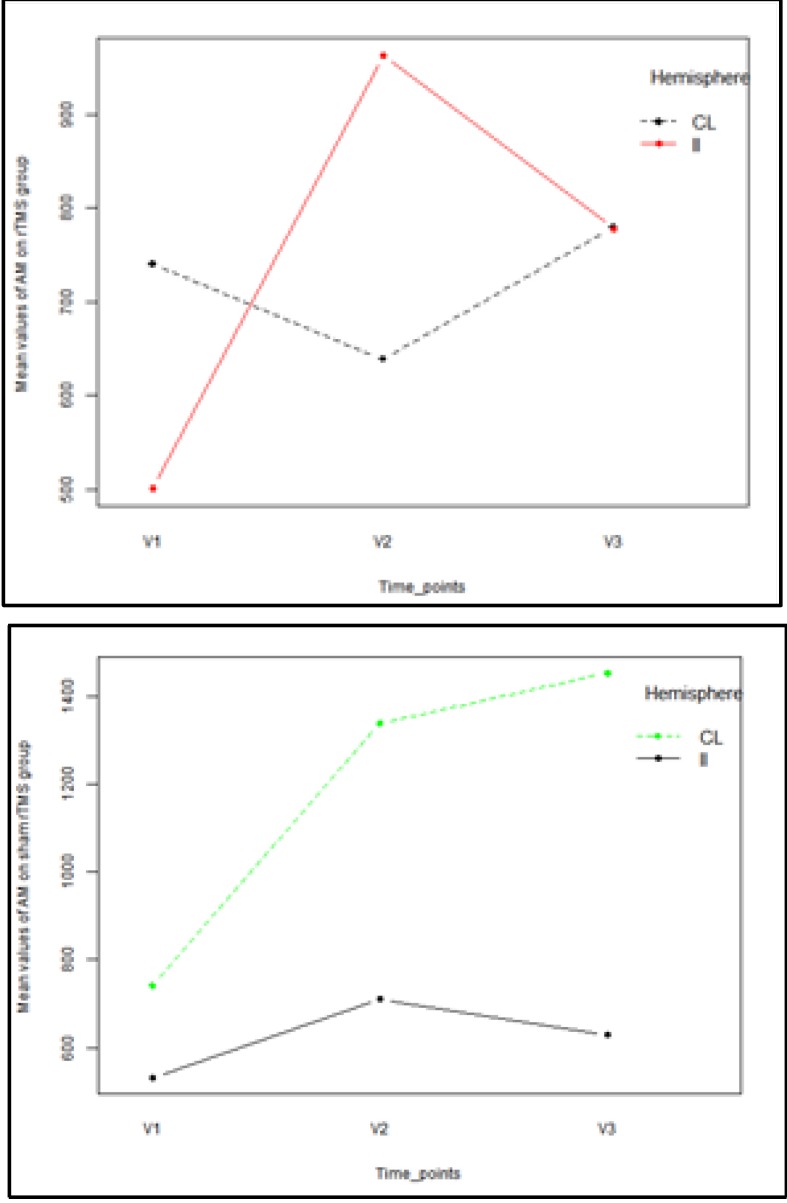
Mean plot of AM parameter on each of interest group

The results showed that there was a trend toward statistical significance in differences between the two hemispheres in AM values [Pillai’s Trace multivariate test: V=0.50; F(3,10)=3.39; p=0.06]. Univariate analysis showed a significant hemisphere effect on AM values only at the V1 timepoint [F(1,12)=7.84; p=0.016]. In both groups, it was observed that the average values of the AM parameter measured in the IL hemisphere were lower than the average AM values in the CL hemisphere [(mean ± standard deviation): 501.01 ± 133.60 versus 740.60 ± 350.41 in the rTMS group and 532.95 ± 211.31 versus 741.55 ± 194.62, respectively]. The results also showed that at the V3 timepoint, the average values of the AM parameter measured in the IL hemisphere were lower than the average AM values in the CL of the rTM group, but this was not statistically significant (p>0.05). In the sham group, the average values of the AM parameter measured in the IL hemisphere were lower than the average AM value in the CL at each timepoint (**Fig. 2**).

According to MANOVA, the group assignment had no effect on the overall mean AM parameter [V=0.14; F(3,10)=0.56; p=0.655], and the change in the mean AM between the hemispheres did not depend on the group assignment [V=0.36; F(3,10)=1.87; p=0.199]. 

**Dynamic changes in Amax parameter**

**Fig. 3:**
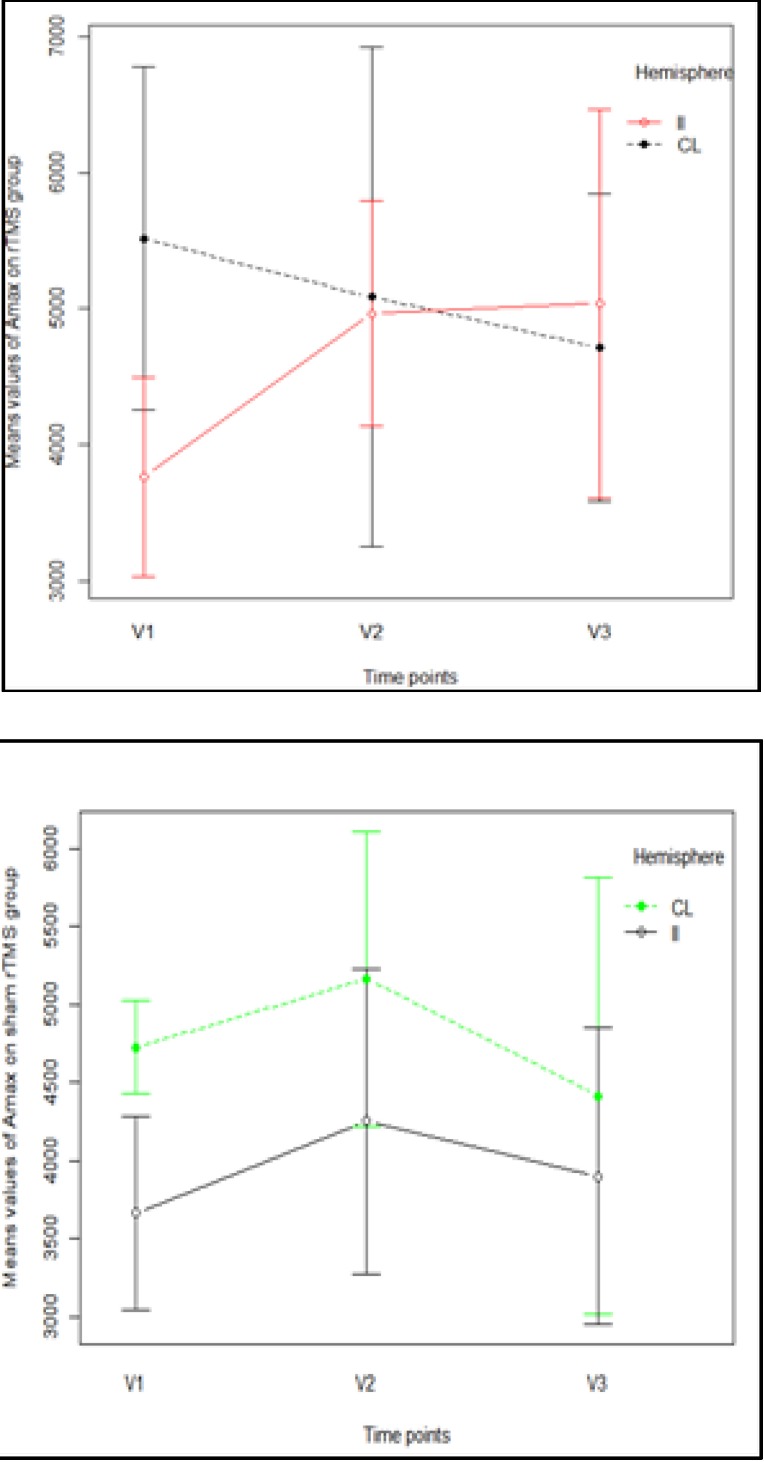
Profile plot of Amax mean values in each of the groups

There was a significant multivariate asymmetry with relation to the type of the hemisphere [Pillai’s Trace multivariate test: V=0.73; F(3,12)=10.66; p=0.001]. Univariate analysis showed a significant hemisphere effect on Amax values at only the V1 timepoint [F(1,14)=8.90; p=0.010]. In both groups, it was observed that the average values of the Amax parameter measured in the IL hemisphere were lower than the average Amax value in the CL hemisphere. In both groups, at the V2 timepoint, the average values of the Amax parameter measured in the CL hemisphere were greater than the average Amax CL values, but this was not statistically significant (p>0.05); while only in the rTMS group were the average values of the Amax parameter measured in the IL hemisphere greater than the average Amax CL values at the V3 timepoint (**Fig. 3**). 

This was not observed in a multivariate group effect [Pillai’s Trace multivariate test: V=5.25; F(3,12)=21.02; p=0.948], nor was a significant interaction observed in a hemisphere × group effect [V=0.41; F(3,12)=2.77; p=0.088].

## Discussion

The dynamics of mapping parameters according to LF-rTMS intervention

In the majority of the patients, at the baseline, we found a decreased excitability in the IL in comparison to the CL side. This result is similar to those of other single-pulse TMS studies of acute/ subacute stroke [**36**,**37**] and is in agreement with fMRI studies that have shown an increased activity in the CL motor area [**14**,**38**]. Furthermore, TMS studies associated the increase in CL hemisphere excitability with an imbalance in transcallosal inhibition, a result that favors the rivalry hypothesis [**39**]. These studies support the idea that the existence of MEP on the affected side after stroke represents a favorable prognostic factor and that the persistence of this effect into the chronic phase of the interhemispheric imbalance has a detrimental effect on rehabilitation [**26**,**40**-**42**]. 

In the real rTMS group, we found that the AM of the IL motor area presented a significant increase from baseline at 45 days, but that after that timepoint, the increase was reduced or even absent. As a result, at 45 days, there was a higher level of excitability in the IL motor cortex in comparison with the CL side, and at 90 days, a rebalance between AM in the two motor areas was observed. In the sham stimulation group, on the IL side, a slight increase as observed in the excitability of the IL motor area, but this was not sufficient to overcome the dominance of the CL side, which was also present at 90 days. Our results are in agreement with the results from other clinical studies of LF-rTMS that have shown that after the intervention, there is a decrease in CL excitability and/ or of transcallosal inhibition that leads to rebalance in excitability between the two hemispheres [**27**,**39**,**43**].

Amax increased in the real rTMS group between V1 and V3 on the IL side, and the increase between V1 and V2 was statistically significant. The values of Amax in the two hemispheres were close to the same at 45 and 90 days. In the sham stimulation group, there was a slight increase in both hemispheres at 45 days and a constant decrease, also in both hemispheres, at 90 days. Although the difference between the two hemispheres was not statistically significant in either group, at V2 and V3, in the sham stimulation group, the difference was much bigger than in the real rTMS group (**Fig. 2**). We analyzed both parameters (AM and Amax) because we observed high variability in amplitudes during mapping, which has also been observed in other single-pulse TMS studies [**44**-**46**]. Explanations for this variability include the presence of spontaneous physiological oscillations in cortical and spinal motoneuron excitability and the interference of TMS stimulation with the excitability of the cortex. This variability might even be increased in patients with acute/ subacute stroke compared to those with chronic stroke or healthy individuals due to altered membrane potentials and ionic imbalance [**47**-**49**]. In light of this variability, we determined that Amean was more reliable than Amax. The evolution of Amean was also more consistent with the evolution of the motor function. 

Our analyses of rMT did not show a specific timing pattern between the 2 hemispheres in these two groups of patients, similar to what has been observed in other studies of MEPs in acute stroke patients [**26**,**50**,**51**]. One possible explanation is that MT is influenced by multiple structural and functional characteristics of both cortical and spinal neurons [**52**]. The mapping area showed a difference between the IL and CL hemispheres in both groups at baseline, with an increased number of RP on the CL side. Only in the real rTMS group did we observe a significant increase in the number of RP from V1 to V3, with the most substantial increase being between V1 and V2. This result is similar to the results described by Freundlieb N. et al. [**53**].

Short and long-term outcomes of motor function after LF-rTMS

The present results suggest that LF-rTMS stimulation improves motor outcome by as early as 45 days, and by 90 days, it favors the rebalance between excitability parameters in the IL and CL motor cortex. However, it does not provide a long-term benefit compared with the sham stimulation. Similar results were found in a clinical study of bilateral transcranial direct current stimulation [**54**], which showed that even when the intervention succeeded in reducing interhemispheric imbalance, the clinical outcome was not improved as much as expected. 

One possible explanation in our case could be the lack of stratification of the patients, who had either cortical or subcortical lesions and displayed a wide range FMA-UE scores, from 18-48 (out of a maximum of 66 points). To support this theory, the bimodal balance-recovery model combines the interhemispheric competition model with ideas of the vicariation model [**55**]. According to this hypothesis, the surviving functions of the motor areas and of the corticospinal tract in the IL hemisphere, as well as the integrity of the brain in the CL hemisphere, play an important role in tipping the scale in the direction of either the beneficial or the detrimental effects that result from CL hemisphere over-excitability. For example, in extensive strokes, where structural preservation is low or even absent, CL hemisphere hyperexcitability may represent the main mechanism of compensation for neural loss. Patients may also develop different connectivity patterns during rehabilitation dependent on whether they experienced a cortical or a subcortical stroke [**56**].

Hypothesis regarding the influence of the evolution of cortical excitability upon rehabilitation

Multiple lines of evidence support the hypothesis that the hyperexcitability of the CL motor area is a functional compensatory mechanism [**20**,**57**-**60**]. On the other hand, it has been shown that the CL side can support, but cannot replace, the affected side. Furthermore, extensive plasticity in the CL motor cortex can be detrimental in the presence of aberrant rewiring [**61**]. Although the role of the CL hemisphere in stroke rehabilitation remains controversial, there is an increasing amount of evidence indicating that the dichotomous view that considers the over-activation of the CL side to be only detrimental represents an important source of disappointment in NIBS trials [**55**].

Interhemispheric functional connectivity (IHC) has been extensively used by fMRI. IHC appears to be decreased in the acute phase of stroke and then increased after the first week. In the chronic phases, IHC is reestablished or may even increase to compensate for damaged anatomical connections [**62**,**63**]. Diffusion tensor imaging (DTI) results have shown that decreased IHC is a consequence of corticospinal tract impairment, which leads to transsynaptic axonal degeneration [**63**]. Because this degenerative process also involves the CL hemisphere **64**], it seems logical to assume that the micro-structural reorganization of the CL side, by processes including axonal sprouting and dendritic branching [**65**], can also affect interhemispheric connectivity and IL hemisphere reorganization [**66**]. In support of this theory is the dynamic evolution of the relationship between IL and CL excitability, which occurs as an over-activation of the CL hemisphere during the acute/ subacute phase and normalization afterwards, at least in cases with a good outcome. The theory that the structural support of synaptic plasticity that is provided by the CL motor area may explain the absence of long-term improvements after LF-rTMS in a heterogeneous group of patients, from the point of view of stroke severity. In the case of a patient with a severe ischemic lesion with little functional or structural preservation in the IL hemisphere, LF-rTMS intervention will have 3 effects: 1) it will diminish the dominance of the CL motor area, which will have a short-term positive impact on motor function, as it was observed in our results at 45 days post-stroke; 2) it will inhibit CL motor area neuronal plasticity, which will have a negative impact on long-term plasticity in the lesioned side, as it was shown by the lack of clinical improvement in our study from V2 to V3; 3) and it will inhibit the functional support of the CL hemisphere. In the case of a patient with a mild lesion, transcallosal connectivity will be supported by the ipsilateral hemisphere, resulting in the inhibition of the CL motor cortex being likely to have a positive effect on neurorehabilitation. 

## Conclusions

This study shows that LF-rTMS enhances motor improvement at 45 days, but not at 90 days, post-stroke. LF-rTMS may also promote an increase in excitability on the affected side at 45 days, compared to the CL side, and a rebalance of interhemispheric excitability at 90 days. There is a need for studies with larger numbers of patients to counteract the high degree of variability observed in amplitudes in stroke patients. Stratified studies using customized rTMS interventions are mandatory to determine whether different types of stimulation fit different types of patients, e.g., cortical versus subcortical stroke, mild motor deficits versus severe motor deficits, and small ischemic lesions versus extensive ischemic lesions. 
